# Expanding the diagnostic spectrum of malignant otitis externa: a case report of proteus infection in a non-immunosuppressed patient

**DOI:** 10.3389/fmed.2025.1577525

**Published:** 2025-07-02

**Authors:** Fabián Darío Arias Rodríguez, Mercedes Larenas, Santiago Paredes, Guillermina Giuliano, Andrés López-Cortés, Juan S. Izquierdo-Condoy

**Affiliations:** ^1^Hospital Zonal General de Agudos Dr. Ricardo Gutierrez, La Plata, Argentina; ^2^Cancer Research Group (CRG), Faculty of Medicine, Universidad de Las Américas, Quito, Ecuador; ^3^One Health Research Group, Universidad de las Américas, Quito, Ecuador

**Keywords:** malignant otitis externa, *Proteus mirabilis*, atypical presentation, targeted antibiotic therapy, non-immunocompromised patient

## Abstract

**Introduction:**

Malignant otitis externa (MOE) is a severe and potentially life-threatening infection of the external auditory canal, most frequently caused by *Pseudomonas aeruginosa*. Although typically seen in immunocompromised individuals—especially those with diabetes mellitus—up to 45% of cases have been reported in non-diabetic patients, highlighting the importance of recognizing atypical presentations. Rare pathogens, such as *Proteus mirabilis*, have been identified in only a few documented cases. Early diagnosis and prompt intervention are essential to prevent serious complications, including osteomyelitis and intracranial extension.

**Objectives:**

This report describes a really uncommon case of malignant otitis externa caused by *P. mirabilis* in a non-diabetic, immunocompetent patient.

**Case presentation:**

A 53-year-old male with no relevant medical history presented with a four-month history of left-sided otorrhea, otalgia, and preauricular pain. Examination and imaging revealed purulent discharge and bone erosion, suggestive of MOE. Empirical treatment with amoxicillin/clavulanic acid was ineffective. Culture identified *Proteus mirabilis*, resistant to multiple antibiotics but sensitive to piperacillin/tazobactam, which was administered with supportive care. After 17 days of intravenous therapy, the patient improved and was discharged on oral ciprofloxacin to complete a three-month course. Follow-up confirmed clinical resolution without recurrence.

**Conclusion:**

This case report highlights an exceptionally rare occurrence of MEO caused by *P. mirabilis* in an immunocompetent, non-diabetic patient. It emphasizes the need to consider uncommon pathogens and atypical clinical profiles in MOE. Early diagnosis, microbiological confirmation, and tailored antimicrobial therapy were critical for favorable outcomes.

## 1 Introduction

Malignant otitis externa (MOE) is a severe and potentially life-threatening infection of the external auditory canal that extends beyond the cutaneous lining, beginning as cellulitis and potentially progressing to chondritis, periostitis, or osteomyelitis. The condition is most commonly caused by *Pseudomonas aeruginosa* and can spread to adjacent soft tissues or even intracranial structures in advanced stages (1, 2). Although MOE is rare, its incidence has increased in at-risk populations—particularly among immunocompromised individuals—with diabetes mellitus reported in approximately 55–95% of cases (2–4).

The clinical presentation of MOE includes intense otalgia, headache, purulent discharge, and edema of the external auditory canal. Facial paralysis, often involving the facial nerve, is a frequent complication and underscores the importance of early diagnosis and timely treatment to prevent progression to severe outcomes such as osteomyelitis and meningitis (5, 6).

Recent studies have reported a rising incidence of MOE in non-diabetic individuals, accounting for 5 to 15% of all cases. For instance, Sideris et al. (7) found that approximately 11% of EOM cases occurred in patients without diabetes, highlighting other possible risk factors such as immunosuppression or foreign body exposure (7, 8). Other large-scale epidemiological studies from the United States and Taiwan have documented even higher proportions, with up to 45% of MOE cases occurring in non-diabetic patients, although diabetes remains strongly associated with MOE (OR = 7.50; 95% CI: 6.22–9.03) (3, 9).

*Proteus mirabilis*, a Gram-negative bacillus commonly associated with urinary tract and wound infections, is an infrequent but noteworthy pathogen in MOE. Compared to *Pseudomonas aeruginosa*, it has been less documented in this context. A study by Arsovic et al. (10) identified *Proteus mirabilis* in only one out of 30 positive cultures from MOE patients, underscoring its rarity and emerging clinical significance (11).

This report describes a really uncommon case of malignant otitis externa caused by *Proteus mirabilis* in a non-diabetic patient.

## 2 Case presentation

A 53-year-old male presented with a progressive four-month history of left-sided otorrhea, otalgia, and retro- and preauricular pain. He had no significant past medical history, including no diagnosis of diabetes mellitus. Prior treatment with amoxicillin/clavulanic acid had yielded no clinical improvement.

**Figure 1:**
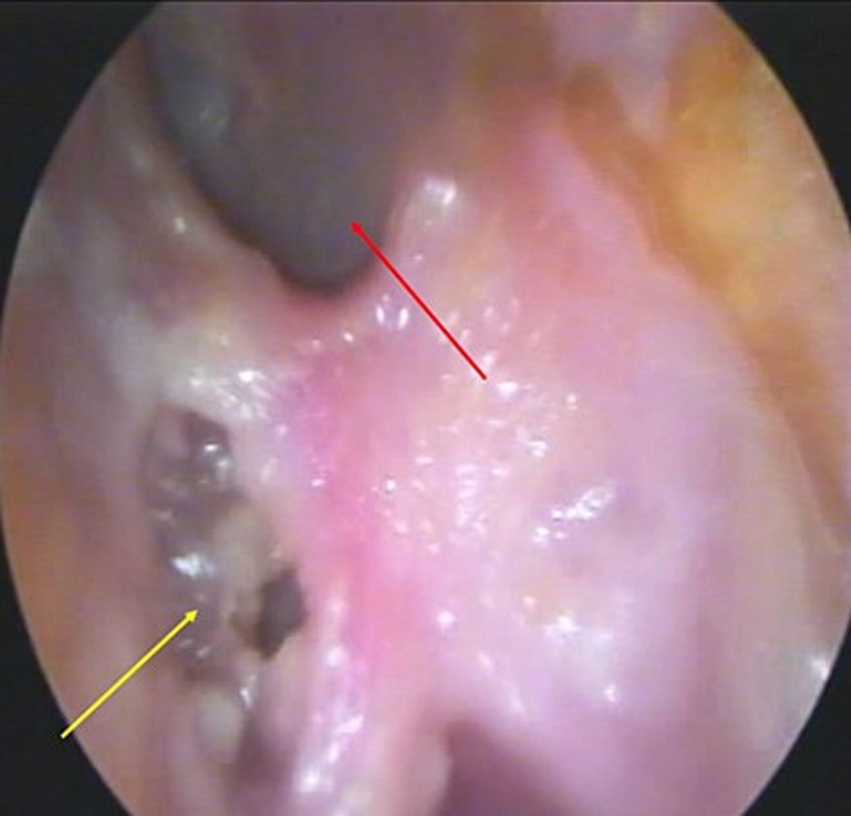
Otoendoscopy of the left ear. Showing bone erosion in the inferior wall of the external auditory canal (yellow arrow) with purulent otorrhea and an intact tympanic membrane (red arrow).

Physical examination revealed signs of cutaneous infection and bone erosion involving the anteroinferior and inferior walls of the external auditory canal (EAC). Otoendoscopy showed abundant yellowish, foul-smelling otorrhea (Figure 1). Based on these findings, the patient was admitted with a presumptive diagnosis of MOE for further evaluation and intravenous therapy.

Initial laboratory investigations revealed leukocytosis of 20,050 cells/μL (reference: 3,600–10,500/μL) with 86% neutrophils, a normal blood glucose level of 91 mg/dL (reference: 70–110 mg/dL), and elevated C-reactive protein (CRP) at 2.4 mg/dL (reference: 0.0–2.0 mg/dL). Serological tests for VDRL and HIV were non-reactive, and blood cultures were negative. Culture of the ear secretion identified *Proteus mirabilis*, resistant to ampicillin, ampicillin/sulbactam, cefazolin, and TMP-SMX but sensitive to azithromycin, cefotaxime, ciprofloxacin, imipenem, meropenem, and piperacillin/tazobactam. Fungal culture was negative (Table 1).

**Table 1:** Summary of laboratory and microbiological findings of patient with MOE.

Parameter	Result	Reference value
Complete blood count		
Hematocrit	42.9%	M: 42–52%, W: 37–47%
Hemoglobin	14.7 g/dL	M: 14–16, W: 12–14 mg/dL
Platelets	315,000 cells/μl	150,000–450,000 cells/uL
Leukocytes	20,050 cells/μl	3,600–10,500 cells/uL
Neutrophils	17,240 cells/μl	1,500–8,500 cells/μl
Lymphocytes	2,205 cells/μl	1,000–4,500 cells/μl
Biochemistry and serology		
Glucose	91 mg/dl	70–110 mg/dL
Creatinine	0.98 mg/dL	0,70–1,30 mg/dL
C-reactive protein	2.4	0,0 – 2,0
VDRL (Syphilis Test)	Non-reactive	
HIV	Non-reactive	
Blood culture	Negative	
Otic secretion culture		
Observation	Scant leukocytes and erythrocytes	
Bacterial culture	*Proteus mirabilis* - Sensitive to ciprofloxacin and piperacillin/tazobactam	
Fungal culture	Negative	

M: male; F: female. Abnormal findings included marked leukocytosis with neutrophilia and a mildly elevated C-reactive protein level. The culture of otic secretions revealed leukocytes and a positive bacterial culture for *Proteus mirabilis*, which was sensitive to ciprofloxacin and piperacillin/tazobactam.

**Figure 2:**
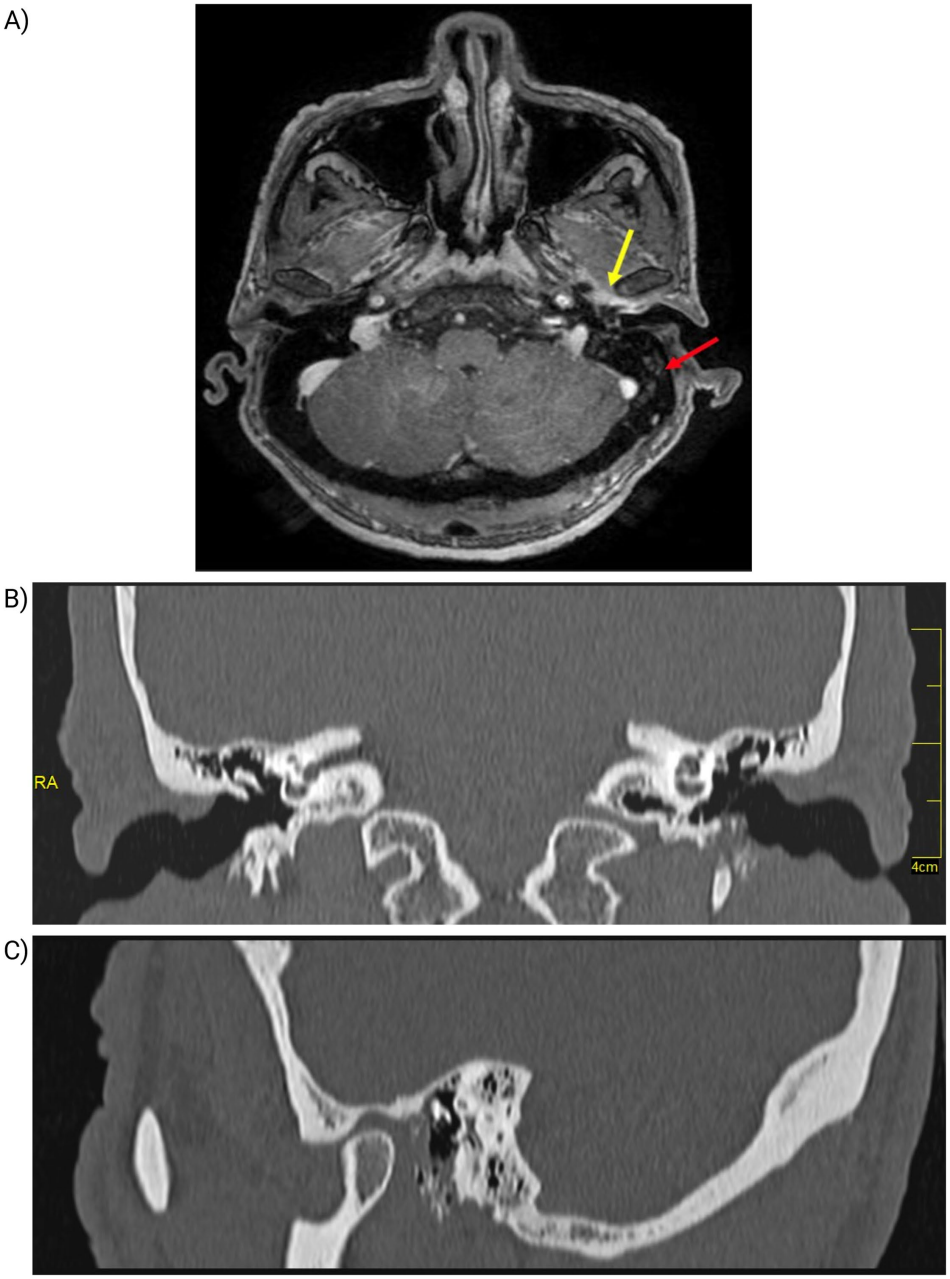
Imaging findings of the left ear. **(A)** Magnetic resonance imaging (MRI) of the left ear. Demonstrating opacification of the mastoid air cells (red arrow) and altered signal intensity at the inferior margin of the external auditory canal (yellow arrow). **(B,C)** Computed Tomography of the Left Ear. **(B)** Coronal CT scan shows bone erosion in the anteroinferior and inferior walls of the external auditory canal (red arrow). **(C)** CT scan shows soft tissue density with bone involvement (yellow arrow).

On the third day of hospitalization, magnetic resonance imaging (MRI) was performed, revealing mastoid cell occupation on the left side, with signal intensity alterations along the lower border of the EAC. A 13 mm × 9 mm fluid-filled lesion in the inner third of the EAC demonstrated peripheral enhancement following gadolinium administration (Figure 2A). As part of the diagnostic workup, a computed tomography (CT) scan was also obtained, confirming soft tissue density in the anteroinferior wall of the EAC, with associated bone erosion—consistent with damage to the underlying bony structures of the anteroinferior EAC wall (Figures 2B,C).

Based on culture sensitivity results, intravenous piperacillin/tazobactam (4.5 g every 6 h) was initiated, along with analgesics and anti-inflammatory agents including dexamethasone, diclofenac, and paracetamol. Multiple aspirations of purulent material were performed during hospitalization, revealing granulation tissue and necrotic debris, which were debrided.

After 7 days of treatment, leukocyte counts declined to 11,700 cells/μL, with normalization of neutrophil levels. Liver and metabolic function tests remained within normal ranges. The patient continued intravenous piperacillin/tazobactam throughout his 17-day hospital stay, showing consistent clinical improvement. At discharge (day 17), he was transitioned to oral ciprofloxacin (500 mg every 12 h) and omeprazole (20 mg daily) to complete a three-month antibiotic regimen. Outpatient follow-up was scheduled biweekly for the first 2 months and monthly thereafter for a total of 6 months.

**Figure 3:**
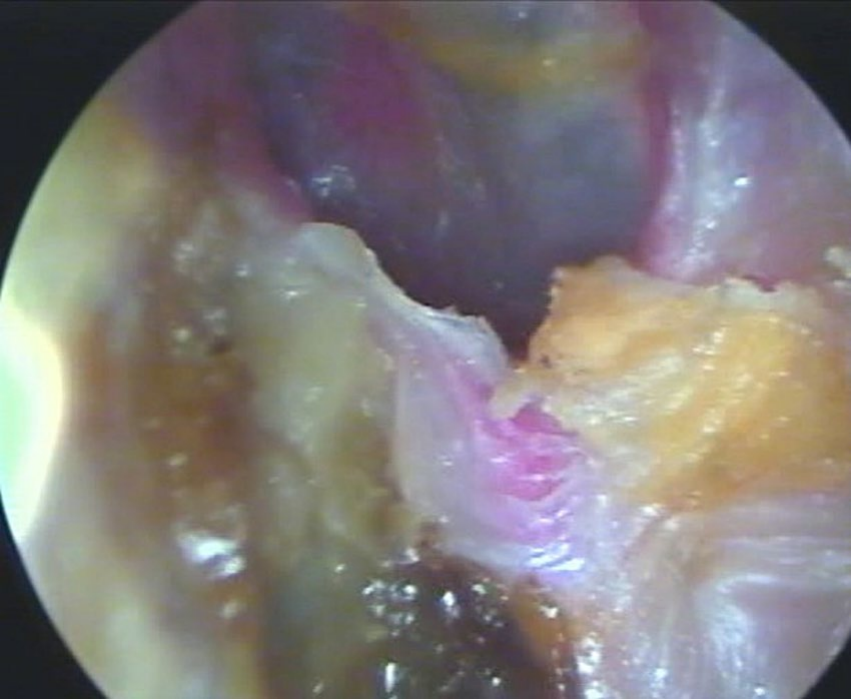
Follow-up otoendoscopy of the left ear. The image shows a dry ear without purulent discharge, cerumen production, and an intact tympanic membrane.

At the three-month follow-up, the patient demonstrated substantial clinical improvement. Repeat otoendoscopy showed near-complete resolution of the infection (Figure 3). Long-term monitoring remains ongoing to ensure full recovery and prevent recurrence. This case report was prepared in accordance with the CARE (CAse REport) guidelines to ensure transparency and completeness in clinical case reporting.

## 3 Discussion

**Figure 4:**
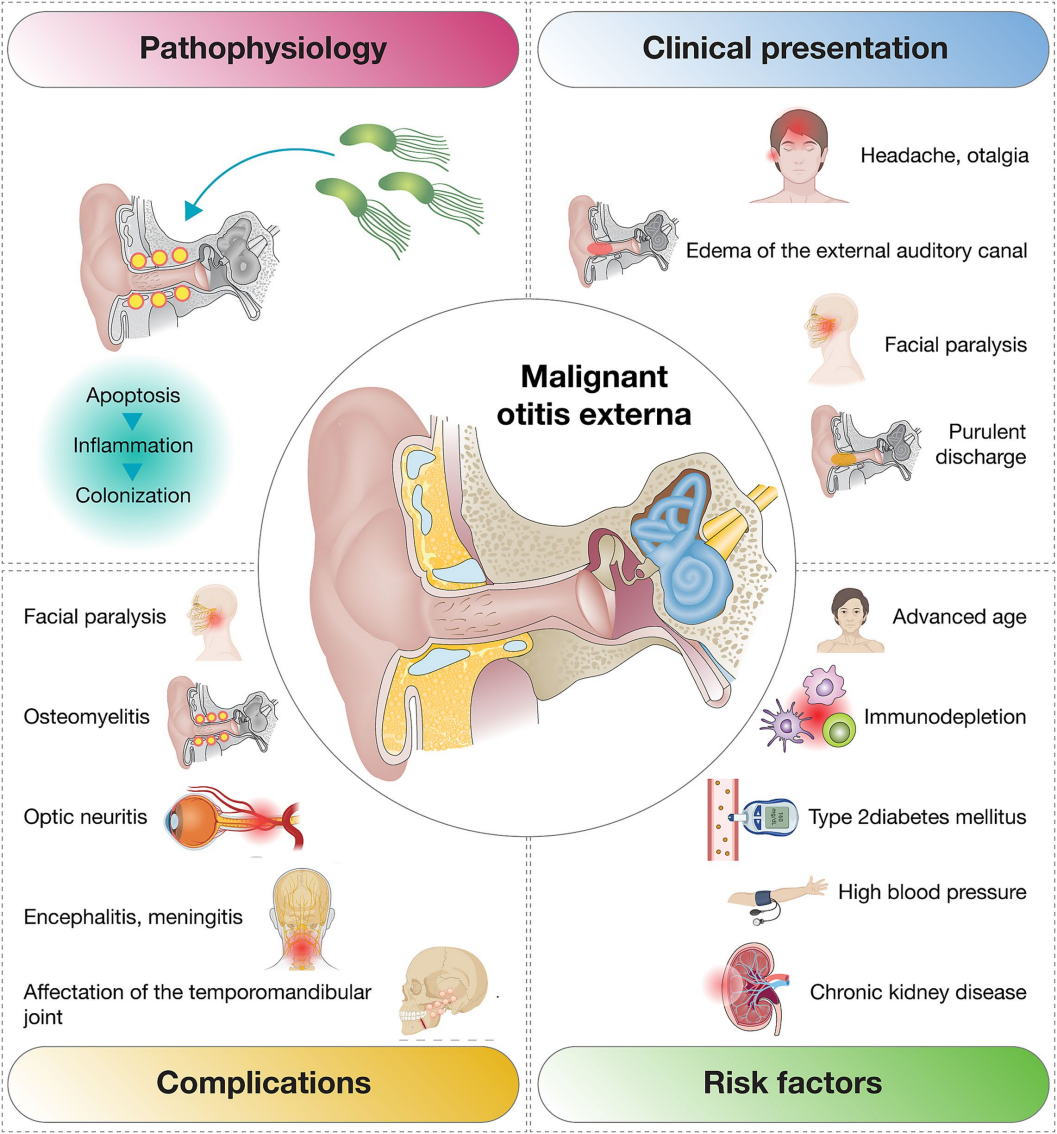
Overview of the most frequent clinical presentations, complications, and risk factors associated with malignant otitis externa.

MOE is a severe infection of the external auditory canal that primarily affects elderly and immunocompromised individuals, with diabetes mellitus being the most common underlying condition associated with its development (7). This case emphasizes the importance of early diagnosis and appropriate treatment, even in atypical presentations. Notably, the patient in this report had no underlying comorbidities, such as diabetes mellitus or other immunosuppressive states (2, 12, 13) (Figure 4).

In this case, the clinical presentation included otalgia and otorrhea in the absence of systemic symptoms, aside from leukocytosis noted on admission. This highlights the need for high clinical suspicion and timely diagnostic intervention to prevent the aggressive progression often seen in MOE (14). The patient presented with persistent, severe otalgia, purulent otorrhea, inflammation of the external auditory canal, and bone necrosis, as confirmed by otoscopic and imaging findings. Both CT and MRI were performed to confirm the diagnosis and assess the extent of bony involvement. Additional diagnostic modalities, such as bone scintigraphy, can also be considered to evaluate skull base involvement when needed (15).

Moreover, inflammatory biomarkers such as the erythrocyte sedimentation rate (ESR) may serve as useful adjuncts in the diagnosis and monitoring of MOE (16). Although ESR was not measured in this case, its inclusion is supported by literature, particularly in patients with suspected systemic inflammatory responses or advanced disease. Future case series and clinical guidelines should consider incorporating ESR as part of the standard diagnostic workup for MOE.

The clinical and radiological features in this case met the major and minor diagnostic criteria proposed by Cohen and Friedman in 1987, reaffirming the enduring relevance of these criteria, especially in resource-limited settings (15).

Given the initial clinical and imaging findings, carcinoma of the external auditory canal was considered as a differential diagnosis. This was based on the lesion’s appearance and imaging characteristics, which raised suspicion for a malignant process. Although no biopsy was performed, the absence of suspicious masses on imaging, the lack of progressive lesion growth, and the favorable response to targeted antibiotic therapy supported a non-neoplastic etiology. Culture of the ear discharge revealed a predominant inflammatory process with no evidence of malignancy, thereby excluding carcinoma and redirecting management toward MOE (10, 15).

The clinical manifestations observed in this case align with those reported by Arsovic et al. (10), who identified otalgia (76%), otorrhea (79%), external auditory canal edema (45%), and granulation tissue (31%) as the primary symptoms among 30 MOE patients. Such consistency underscores the importance of recognizing these hallmark symptoms in clinical practice.

Identification of the causative pathogen is critical for achieving favorable outcomes in MOE. While *Pseudomonas aeruginosa* is implicated in over 95% of cases, *Aspergillus* accounts for a smaller proportion (11). However, rare pathogens like *Proteus mirabilis* have been documented in isolated cases. For instance, Arsovic et al. (10) reported *P. mirabilis* in only one out of 30 MOE cases, a finding corroborated by AlSharhan et al. (17), who identified *P. mirabilis* as the causative agent in just one of 28 patients with otogenic skull base osteomyelitis. The identification of *P. mirabilis* in this patient highlights the need to consider uncommon pathogens in MOE, particularly in atypical presentations or non-immunosuppressed patients.

Antimicrobial resistance presents an increasing challenge in the management of MOE, underscoring the necessity of obtaining cultures and antibiotic susceptibility testing to guide treatment (18). In this case, the *P. mirabilis* was resistant to several agents, including ampicillin (a resistance rate reported in 10–20% of isolates), but was sensitivity to piperacillin-tazobactam, ciprofloxacin, and other agents, allowing targeted treatment. Multidisciplinary management, involving otorhinolaryngologists and infectious disease specialists, proved instrumental in achieving a favorable outcome (19). Intravenous piperacillin-tazobactam combined with serial aspiration of purulent discharge successfully controlled the infection, consistent with literature advocating aggressive antibiotic therapy to prevent complications such as sepsis or temporal bone osteomyelitis (17, 20). Additional interventions, such as surgical debridement or hyperbaric oxygen therapy, are typically reserved for refractory cases or poor responses to medical treatment (21–23).

This case broadens the diagnostic and therapeutic understanding of MOE, particularly in non-immunosuppressed patients. It underscores the importance of continuing education for healthcare professionals to improve awareness of MOE, including its risk factors, clinical presentation, and management strategies in atypical scenarios. Enhanced awareness could improve early diagnosis, guide tailored treatment, and ultimately lead to better outcomes for affected patients.

## 4 Conclusion

This case report describes an exceptionally rare instance of MEO caused by *P. mirabilis* in a non-immunocompromised patient, broadening the understanding of the clinical spectrum and etiological diversity of this condition. It underscores the need to consider atypical pathogens in cases where traditional risk factors, such as diabetes mellitus or immunosuppression, are absent.

The favorable outcome in this patient highlights the pivotal role of early diagnosis, thorough microbiological evaluation, and tailored antibiotic therapy in the successful management of MEO. The infection was effectively controlled through the use of broad-spectrum antibiotics, selected based on culture and sensitivity testing, combined with supportive interventions such as serial aspiration of purulent secretions. This case emphasizes the importance of a comprehensive and individualized approach to managing this potentially life-threatening condition.

## Data Availability

The original contributions presented in the study are included in the article/supplementary material, further inquiries can be directed to the corresponding author.

## References

[1] Sando I Harada T Okano Y Saito R Caparosa RJ. Temporal bone histopathology of necrotizing external otitis. A case report. Ann Otol Rhinol Laryngol. (1981) 90:109–15. doi: 10.1177/000348948109000203 7224508

[2] Treviño González JL Reyes Suárez LL Hernández de León JE. Malignant otitis externa: an updated review. Am J Otolaryngol. (2021) 42:102894. doi: 10.1016/j.amjoto.2020.102894 33429178

[3] Yang T-H Xirasagar S Cheng Y-F Wu C-S Kao Y-W Shia B-C. Malignant otitis externa is associated with diabetes: a population-based case-control study. Ann Otol Rhinol Laryngol. (2020) 129:585–90. doi: 10.1177/0003489419901139 31976744

[4] Hodgson SH Sinclair VJ Arwyn-Jones J Oh K Nucken K Perenyei M. Characteristics, management and outcome of a large necrotising otitis externa case series: need for standardised case definition. J Laryngol Otol. (2022) 136:604–10. doi: 10.1017/S002221512100462X 35042578 PMC9257435

[5] Di Lullo AM Russo C Piroli P Petti A Capriglione P Cantone E. Malignant otitis external: our experience and literature review. Am J Case Rep. (2020) 21:e925060. doi: 10.12659/AJCR.925060 32808601 PMC7458700

[6] Mahdyoun P Pulcini C Gahide I Raffaelli C Savoldelli C Castillo L. Necrotizing otitis externa: a systematic review. Otol Neurotol. (2013) 34:620–9. doi: 10.1097/MAO.0b013e3182804aee 23598690

[7] Sideris G Latzonis J Avgeri C Malamas V Delides A Nikolopoulos T. A different era for malignant otitis externa: the non-diabetic and non-immunocompromised patients. J Int Adv Otol. (2022) 18:20–4. doi: 10.5152/iao.2022.21313 35193841 PMC9449702

[8] Glikson E Sagiv D Wolf M Shapira Y. Necrotizing otitis externa: diagnosis, treatment, and outcome in a case series. Diagn Microbiol Infect Dis. (2017) 87:74–8. doi: 10.1016/j.diagmicrobio.2016.10.017 27806892

[9] Sylvester MJ Sanghvi S Patel VM Eloy JA Ying Y-LM. Malignant otitis externa hospitalizations: analysis of patient characteristics. Laryngoscope. (2017) 127:2328–36. doi: 10.1002/lary.26401 27882553

[10] Arsovic N Radivojevic N Jesic S Babac S Cvorovic L Dudvarski Z. Malignant otitis externa: causes for various treatment responses. J Int Adv Otol. (2020) 16:98–103. doi: 10.5152/iao.2020.7709 32209516 PMC7224427

[11] Muraleedharan M Keshri A Rao RN Mehrotra A Das KK Dubey A. Aspergillus infections of lateral skull base: a case series. Eur Arch Otorrinolaringol. (2024) 281:1221–9. doi: 10.1007/s00405-023-08218-z 37668755

[12] Liu X-L Peng H Mo T-T Liang Y. Malignant otitis externa in a healthy non-diabetic patient. Eur Arch Otorrinolaringol. (2016) 273:2261–5. doi: 10.1007/s00405-015-3738-y 26233245

[13] Unadkat S Kanzara T Watters G. Necrotising otitis externa in the immunocompetent patient: case series. J Laryngol Otol. (2018) 132:71–4. doi: 10.1017/S0022215117002237 29173202

[14] Ali T Meade K Anari S ElBadawey MR Zammit-Maempel I. Malignant otitis externa: case series. J Laryngol Otol. (2010) 124:846–51. doi: 10.1017/S0022215110000691 20388240

[15] Cohen D Friedman P. The diagnostic criteria of malignant external otitis. J Laryngol Otol. (1987) 101:216–21. doi: 10.1017/s0022215100101562 3106547

[16] Al Aaraj MS Kelley C. Necrotizing (Malignant) Otitis Externa. Treasure Island, FL: StatPearls Publishing (2025). 32310598

[17] AlSharhan SS Alwazzeh MJ Alshrefy AJ Albahrani NA Telmesani LS AA AG. Microbial spectrum, management challenges, and outcome in patients with otogenic skull base osteomyelitis. Infez Med. (2024) 32:340–51. doi: 10.53854/liim-3203-8 39282550 PMC11392546

[18] Frost J Samson AD. Standardised treatment protocol for necrotizing otitis externa: retrospective case series and systematic literature review. J Glob Antimicrob Resist. (2021) 26:266–71. doi: 10.1016/j.jgar.2021.06.015 34273591

[19] Dhariwal A Manjaly JG Patel B Morris-Jones S David K Khetarpal P. Management and clinical outcomes of 37 patients with necrotizing otitis externa: retrospective review of a standardized 6-week treatment pathway. J Int Adv Otol. (2023) 19:223–7. doi: 10.5152/iao.2023.22637 37272640 PMC10331632

[20] Lambor DV Das CP Goel HC Tiwari M Lambor SD Fegade MV. Necrotising otitis externa: clinical profile and management protocol. J Laryngol Otol. (2013) 127:1071–7. doi: 10.1017/S0022215113002259 24169084

[21] Álvarez-Álvarez M Benito-Orejas JI Carranza-Calleja MA Cámara-Arnaz JA Viveros-Díez P Santos-Pérez J. Otitis externa maligna. Experiencia a lo largo de 25 años en un hospital de tercer nivel. *Revista*. ORL. (2023) 14:63. doi: 10.14201/orl.31063

[22] Balcázar Rincón LE Ramírez Alcántara YL. Otitis externa maligna. Rev Esp Méd Quirúrg. (2014) 19:104–9.

[23] Byun YJ Patel J Nguyen SA Lambert PR. Necrotizing otitis externa: a systematic review and analysis of changing trends. Otol Neurotol. (2020) 41:1004–11. doi: 10.1097/MAO.0000000000002723 32569149

